# Job demands, resources, and task performance in Chinese social workers: Roles of burnout and work engagement

**DOI:** 10.3389/fpubh.2022.908921

**Published:** 2022-07-19

**Authors:** Bin Tu, Xiaoting Luo, Sophie Sitar, Chienchung Huang

**Affiliations:** ^1^Guangdong Research Center for NPO, Guangdong University of Foreign Studies, Guangzhou, China; ^2^School of Public Administration, Guangdong University of Foreign Studies, Guangzhou, China; ^3^Law School, Rutgers, The State University of New Jersey, Newark, NJ, United States; ^4^School of Social Work, Rutgers, The State University of New Jersey, New Brunswick, NJ, United States

**Keywords:** burnout, job demands, job resources, task performance, social workers, work engagement

## Abstract

Social work is a rapidly developing occupation in China. In the early 2000s, there were merely a few hundred thousand social workers, but by 2020 there were over 1.5 million social workers in the field. However, research has indicated these social workers are also experiencing record high burnout and turnover rates. Thus, researchers have started to question the work engagement and task performance factors that could be contributing to these increasing rates. This study uses the Job Demands and Resources (JD-R) Theory to understand how 537 social workers from Guangzhou, China are impacted by burnout and how it influences work engagement and task performance. The results show JD-R directly affect task performance through burnout and work engagement *via* a dual process. First, job demands were associated with high burnout and low work engagement, which both were found to lead to low task performance. Second, job resources were related to low burnout rates and high work engagement, both of which were associated with high task performance. These findings call for healthcare interventions to reduce burnout and workplace policy changes to promote work engagement to support task performance in social workers in China. These factors can each have a crucial impact on the public health of both the affected social workers and the vulnerable clients these social workers serve.

## Introduction

Throughout the last century, the Chinese social work industry has grown exponentially in tandem with China's increasing economy (1, 2). However, the past decade has brought about the most change yet. In 2010, there were an estimated 0.2 million social workers, but by 2020 there were an estimated 1.5 million workers within the field (1, 3, 4). This rapid but massive influx of social workers highlights the quick expansion of the social work field, and of the increasing impact social workers have had on public health, given social work agencies began serving largely disadvantaged communities with poor healthcare access and services (5, 6).

However, it is important to note social work has faces several challenges in development. First, it has not progressed evenly throughout the various regions of China. For example, in more urban cities such as Shenzhen, the social work industry has blossomed due to the greater access to resources. Comparatively, rural regions in western China such as Guizhou have not witnessed these same types of social work changes (7). Second, the professional identity has not yet been fully established and often the public mistakenly perceives social workers to be volunteers. Without nationwide recognition, social workers are continuously deprived of professional respect, which influences their capacity to implement professional, helping work (8, 9).

Third, there is a knowledge gap between what social work education programs teach and how social workers practice in the real world. For example, within Chinese culture certain healthcare concepts, such as mental health, are stigmatized and thus widely underreported, underassessed, and undereducated in professional education programs (10). Social workers based in China frequently work with vulnerable populations who suffer from mental health disorders, but often social workers underdiagnose or misdiagnose clients because of a lack of formal education on mental health and cultural stigma (10). This disconnect often leaves social workers feeling unprepared and frustrated when entering into the professional work setting (11–13). Moreover, these gaps perpetuate a major mental health crisis.

Additionally, the tasks of frontline social workers in China are incredibly comprehensive. Social workers are generally required to perform administrative work, client visits and contacts, case work and management, group work, program planning and implementation, community networking, evaluation, supervision, and training (14, 15). Because many social work tasks are related to health and social inequity, social workers and public health practitioners share the collective goals of delivering quality services to various vulnerable populations in need (5, 16). Like many public health practitioners, social workers often work overtime and during weekends because of the extensive clients' needs. Moreover, many social workers are using this weekend time to fulfill heavy, government-required administrative tasks that stem from social work service purchasing agreements (11, 17, 18). Finally, social workers in China are often underpaid [on average salaries range from 3,000 to 5,000 Yuan ($500–800 USD) per month] (9, 14) and have few promotional opportunities (9, 11).

Moreover, these increasing professional challenges have drastically increased high burnout and turnover amongst social workers in China (2, 8, 9, 19–21). For instance, 25% of social workers based in Guangzhou quit social work in 2014 (22). Similarly, by 2013 Shenzhen social workers were experiencing a turnover rate well over 22% and by 2015 18.08% of social workers based in Shenzhen had quit their jobs (23). These numbers were well over 10% of what they had been a decade earlier. Additionally, research also suggests burnout and turnover rates have become so popular that 90% of Chinese social work students did not intend to enter the social work industry after graduation (24).

Cross-cultural research has shown a strong association between burnout and turnover rates (18, 25, 26), especially amongst social workers in China (19, 20, 27). Given this strong relationship, it is imperative to acknowledge high rates of burnout and turnover can impose negative consequences for social work practitioners and subsequent client populations. For example, social workers who are experiencing burnout may struggle to provide quality client services. This then creates a system of unethical practices and imposes risks of harming vulnerable clients. Moreover, large turnover rates may mean agencies are understaffed so social workers are forced to take on additional job demands and workloads. These extra work pressures and stressors can lead to more burnout, which perpetuates the cycle of high turnover within the industry.

In addition, studies have shown that burnout significantly affects workers' physical health (28–30) and mental health (31–35). Social workers specifically who experience high rates of burnout and work stress are at significantly higher risk for morbidity (24). For example, von Känel et al. (30) found significant associations between burnout and physical health such as high blood pressure, chronic somatic symptom disorder, and lung disease amongst 5,671 respondents in Switzerland. Additionally, Capone and Petrillo (31) found job burnout was significantly linked to depression (*r* = 0.42) amongst a sample of 285 high school teachers. Likewise, Xie et al. (35) found burnout significantly increased psychological distress (Beta = 0.46) amongst a sample of 897 social workers based in Chengdu, China. Thus, burnout is an important factor of health outcomes for social workers, as well as other professionals in China and beyond (28, 30, 32, 34, 35).

### Research gaps and highlights

As burnout and turnover rates continue to rise amongst social workers, there have been concerns and research gaps on whether social workers are able to maintain work engagement, complete task performance, and sustain quality services within social work agencies, community centers, healthcare centers, and alternative social welfare agencies (3, 9). First, this research paper seeks to utilize the Job Demands and Resources (JD-R) Theory to fill this research gap by examining how job demands (JD) and job resources (JR) affect task performance of Chinese social workers and to understand if burnout and work engagement mediate this relationship. Highlights of this study include an effort to: (1) help spread awareness of the type of work conditions Chinese social workers experience, (2) shed light on the effects burnout and work engagement have on task performance amongst social workers in China, and (3) expand information on how the JD-R theory applies to social workers within China.

Second, this paper seeks to raise awareness of the ethical and public health concerns of this rising social work burnout problem. Social workers, especially those based in China, typically work with vulnerable populations who require significant hands-on services. However, if social workers are experiencing burnout and are disengaged with their work, social workers may be unable to fulfill their ethical duties to zealously serve the clients (36). Thus, clients may have become at-risk for experiencing harm, malpractice, or a lack of resources/support that is available. Given these rising harms to both social workers and clients, this paper seeks to investigate factors of burnout and work engagement that effect social workers to limit this growing healthcare crisis (6, 16).

## The job demands-resources theory

The JD-R Theory posits JD and JR each effect the job performance, health, and overall wellbeing of working employees (37, 38). JD are the physical, social, or organizational job features that require an employee to exert prolonged physical or mental efforts. Additionally, JD typically impose physiological costs, such as fatigue and debilitation. JR are the job features that help facilitate work achievements, mitigate any psychological costs of JD, and encourage personal development (39).

Together, JD and JR affect burnout, work engagement, and health outcomes through two different processes: (1) the health-impairment process (also known as the energy-driven process) and (2) the motivation-driven process. In the health-impairment process, JD are physical and emotional stressors that gradually deplete social workers energy. Subsequently this depletion can lead to high burnout, low work engagement and performance, and poor health outcomes such as chronic somatic symptom disorder and depression (30, 31, 38, 40). In contrast, in the motivation-driven process, JR encourage and support employees in their efforts to meet work responsibilities, which increases work engagement, reduces burnout, and improves various job and health outcomes (33, 35, 38, 41, 42).

*Hypothesis 1: JD are positively associated with burnout*
*Hypothesis 2: JD are negatively associated with work engagement*
*Hypothesis 3: JR are negatively associated with burnout*
*Hypothesis 4: JR are positively associated with work engagement*

### Burnout and work engagement

Burnout is a psychological condition that can create emotional exhaustion, a feeling of being outside one's body, and a reduced sense of personal accomplishment, especially during challenging work situations (43). Thus, burnout is considered an occupational threat to professionals in various human service industries (44, 45) such as social workers (35, 46). In contrast, work engagement is a positive, fulfilling, work-related state of mind that is characterized by vigor and dedication (42, 47). Burnout is an important predictor of physical and mental health outcomes, (28, 30, 31, 33, 35) while work engagement is a crucial factor of motivational outcomes (38, 40).

### Task performance

Task performance can be understood as the efficiency with which an employee performs assignments that contribute to core job responsibilities. Additionally, task performance measures the employee's success at delivering specific work outcomes, as well as quality and quantity work (48, 49). It is critical to examine burnout, work engagement, and task performance through the lens of JD-R Theory (11, 21, 39) as numerous researchers have discovered JD and JR have direct effects on task performance, as well as indirect effects through burnout and work engagement (18, 21, 35, 38). Task performance is negatively affected by JD and burnout (50–53). For example, one study by Bakker and Heuven (50) found that of 108 nurses and 101 police officers, burnout negatively affected both sample's task performance (beta = −0.32 in nurse sample and −0.35 in police sample). Likewise, Dyrbye et al. (51) found that amongst a national sample of U.S. nurses, those who were experiencing burnout were also more likely to have poor task performance (*n* = 3,098).

In contrast, task performance is positively affected by JR and work engagement (52, 53). One study by Halbesleben and Wheeler (52) found that of 587 employees from various industries and occupations, work engagement was associated with high task performance amongst self-reported, supervisor-rated, and coworker-rated performance. Additionally, Song et al. (53) also found that amongst a sample of 481 Korean teachers, work engagement was positively correlated with task performance (beta = 0.23).

*Hypothesis 5: JD are negatively associated with task performance*
*Hypothesis 6: JR are positively associated with task performance*
*Hypothesis 7: Burnout is negatively associated with task performance*
*Hypothesis 8: Work engagement is positively associated with task performance*

In addition to the extensive research conducted on task performance, the JD-R Theory has also been widely utilized to understand how burnout, stress, work engagement, health, and task performance affect work outcomes (29–31, 35, 45, 50, 51, 54). Studies have shown JD can substantially influence the health-impairment process which in turn can cause burnout, low work engagement, and poor health outcomes. Meanwhile, JR, through a motivation process, can be a significant protective factor against burnout and can even increase work engagement.

Despite the volume of JD-R research in this area, few studies have focused on whether burnout and work engagement mediate the effects of JD-R on task performance of social workers in China. This is an important gap, especially considering the serious and negative health outcomes stemming from social worker burnout and how these effects spill-over onto the clients they serve. Thus, this study fills the research void. We use the JD-R Theory to examine the effects of JD-R on task performance and seek to understand if there are any relationships mediated by burnout and work engagement amongst a sample of social workers in China.

*Hypothesis 9: Burnout and work engagement mediate the effects between JD-R and task performance*

## Methods

### Data and sample

Our study consisted of an online anonymous survey which was administered to working social workers in Guangzhou, China. Guangzhou is the capital of Guangdong province and has undergone rapid social work developments within the last decade (55). In 2017, Guangzhou policymakers created street level social work service stations to expand social services offered within the local communities. Each of these stations are operated by 20 social workers and 14 of them are front-line social workers, whom offer immediate services/referrals for housing, healthcare, education, and employment. For example, the healthcare services include health management at elderly daycare centers and working with local community health service centers to provide education on disease prevention.

We randomly selected 54 of the 180 Guangzhou service stations to sample, and emailed survey participation links to the front-line social workers at each site on September 15, 2021. We then sent follow-up notices 7 and 14 days later to remind participants to complete the initial survey. Out of 756 social workers we initially emailed (54 ^*^ 14), 537 social workers answered the online survey by October 10, 2021. Thus, the response rate was 71%. Each participant was notified of their right to informed consent prior to their participation in the survey. Each participant was also informed their participation was voluntary and of their ability to stop the survey at any time. The research protocol was also approved by the research review committee at one of the co-authors' university in China. The demographics show a majority of our sample identified as female (84.5%) and were never married (54.2%). The average age of the sample was 29.3 (SD = 6.3). Over half of the sample had at least a college degree (54.2%). Additionally, the mean income and work experience of social workers were 4,355 yuan (~$647 USD) monthly and 3.7 years, respectively.

### Measures

First, we measured task performance using Goodman and Svyantek's (56) 9-item task performance scale. Numerous studies have verified this scale's validity, psychometric soundness, and reliability (56–58). The scale assessed task performance through a series of questions that assess whether the worker feels he/she is able to “achieve the objectives of the job” and “plan and organize to achieve objectives and meet deadlines” for example. Each item was rated on a seven-point Likert scale, ranging from 0 to 6. High scores meant the item was completely characteristic of the employee, while a low score meant it was not characteristic of the employee. We averaged a mean score of all 9 items as the task performance score. The Cronbach's alpha of task performance was 0.94 in this study.

Second, we assessed for work engagement using a short form of the Utrecht Work Engagement Scale (UWES-9) (59). The short form version of this scale has been verified by numerous studies for its validity, reliability, and soundness (59–61). The UWES-9 includes nine items to gauge the three dimensions of work engagement: vigor, dedication, and absorption. Each has three items. Initially, participants were tasked with answering opinion questions about their jobs. Exampled questions were: “At my work, I feel bursting with energy” (vigor), “I am enthusiastic about my job” (dedication), and “I am immersed in my work” (absorption). Each item was assessed along a 7-point Likert scale ranging from 0 (“never”) to 6 (“always”). The overall work engagement score was an average mean score of all 9 items. The Cronbach's alpha of work engagement was 0.95 in this study.

Third, we measured burnout using the Oldenburg Burnout Inventory (OBI) (62) which has been utilized frequently to study burnout amongst working professionals on an international scale (39, 63, 64). The OBI survey consisted of 16 items which assessed for exhaustion (8 items) and disengagement from work (8 items) through questions such as: “It happens more and more often that I talk about my work in a negative way”. Questions geared toward studying exhaustion sought to understand the intense physical, emotional, and mental pressures each employee experiences at work, while questions geared toward disengagement examined how the employee distanced himself from his work generally, work object, and/or work content (62). Each item was measured in 4 categories (1 = strongly disagree to 4 = strongly agree). We reversed positively worded items so that high scores represented high burnout. We averaged mean score of all 16 items as the burnout score. The Cronbach's alpha of the scale was 0.85 in our study.

Finally, we measured JD-R using a multidimensional scale from Lequeurre et al.'s (65) Questionnaire sur les Ressources et Contraintes Professionnelles (QRCP). This scale focused on three dimensions of JD– workload, emotional workload, changes in the tasks, –and three areas of JR– relationships with colleagues, relationships with supervisor, and information. Workload refers to the sense of having an extensive quantity of work to complete in minimal time, while emotional workload refers to the JD that require participants to expend emotional energy. Changes in the tasks refers to the participants difficulty within their job functioning when there are changes in agency job roles and responsibilities. Relationships with colleagues concerns the team atmosphere, including whether a respondent can rely on co-workers for help and social support. Relationships with supervisors describes the rapport between a participant and their superior. Lastly, information refers to the participant's access to feedback on his/her work performance from supervisors or co-workers. Lequeurre et al. (65) used four items to measure each dimension. The Cronbach's alpha was high, above 0.80 for each dimension. Each item was also rated according to a 7-point Likert scale with scores ranging from 1 (“never”) to 7 (“always”). The higher the score per item, the higher the level of JD or JR was present. The Cronbach's alpha was 0.85 for all 24 items and were 0.83 and 0.93 for JD and JR in this study. Finally, we calculated JD and JR scores by averaging the item responses under each scale. Appendix 1 lists all scale items used in this study.

### Analytical approach

**Figure 1:**
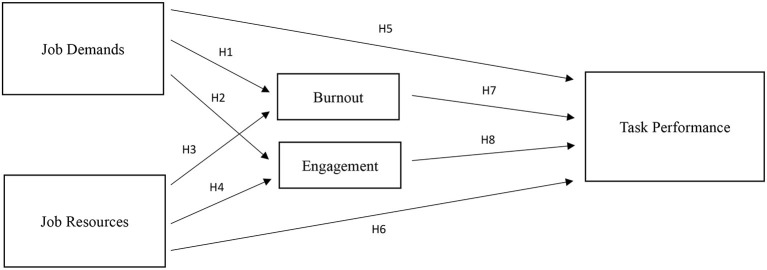
Conceptual model.

Based on the JD-R Theory, we developed a conceptual model, as shown in Figure 1. The figure posits JD and JR directly and indirectly affect task performance because of their effects on burnout and work engagement. In addition, burnout and work engagement also have their own, separate direct effects on task performance.

We conducted analytical analyses using STATA software 16.0. Initially we sought to examine the participants' demographics to determine if there were correlations amongst all the variables. Second, we generated a structural equation modeling (SEM) analysis, to understand the direct and indirect effects of JD-R (the independent variable) on task performance through the hypothesized mediators, burnout, and work engagement using a maximum likelihood method of estimation. We also conducted regression analyses with extensive covariates, including personal characteristics. The results from the regression analyses are similar to those reported here. Results of regression analyses are not provided within this study but can be provided upon request. The common method variance analysis was performed, and the results showed that only 27% of the variance shared by JD-R, work engagement, burnout, and task performance items, suggesting common method variance was not an issue in the data.

## Results

Although the scales used in this study were all from published scales that show verified reliability and validity in literature, it is not clear the extent of the reliability and validity of these scales for Chinese social workers in this study. We conducted confirmatory factor analysis for all scales and the results were presented in Appendix 2. Overall, all scales show acceptable reliability, however, the factor loadings of certain items in burnout and job demands, particularly in emotional workload and changes in tasks dimensions, were low. We removed items with factor loadings <0.50 (66, 67). As a results, burnout items reduced from 16 to 15, and job demand items reduced from 12 to 9. We used the new burnout and job demand scales to conduct the analysis. The results based on original scales, available upon requested, were not significantly different from the ones using new scales.

Table 1 presents the descriptive statistics and correlation of key variables. The sample had an average task performance score of 4.0, which ranged from 0.6 to 6.0. The average work engagement score was 3.5, which ranged from 0.0 to 6.0, while the average score for burnout was 2.5, from a range of 1.1–3.9. Overall, the sample reported moderate task performance with above means work engagement and burnout. The sample also reported an average 5.0 for JD, but answers ranged from 2.0 to 7.0. Additionally, the sample reported an average 5.2 for JR, but answers ranged from 2.2 to 7.0. These results suggest the participants experienced relatively high JD at work but simultaneously received high assistance at work.

**Table 1:** Descriptive statistics and correlations of key variables.

	**Mean (SD)**	**1**	**2**	**3**	**4**	**5**
1. Task performance (0–6)	4.0 (0.9)	–				
2. Work engagement (0–6)	3.5 (1.3)	0.58***	–			
3. Burnout (1–4)	2.5 (0.4)	−0.37***	−0.60***	–		
4. Job demands (1–7)	5.0 (0.8)	0.00	−0.19***	0.51***	–	
5. Job resources (1–7)	5.2 (0.7)	0.45***	0.45***	−0.30***	0.06	–

N = 537; ^***^p <0.001.

The Pearson's correlation analysis results were largely compatible with our hypotheses. As displayed in Table 1, JD were positively correlated with burnout (*r* = 0.51, *p* < 0.001) and negatively correlated with work engagement (*r* = −0.19, *p* < 0.001). Concurrently, JR were negative corelated with burnout (*r* = −0.30, *p* < 0.001) and positively corelated with work engagement (*r* = 0.45, *p* < 0.001). JR were also positively correlated with task performance (*r* = 0.45, *p* < 0.001). Work engagement was also positively correlated with task performance (*r* = 0.58, *p* < 0.001), while burnout was negatively correlated with the task performance (*r* = −0.37, *p* < 0.001).

**Figure 2:**
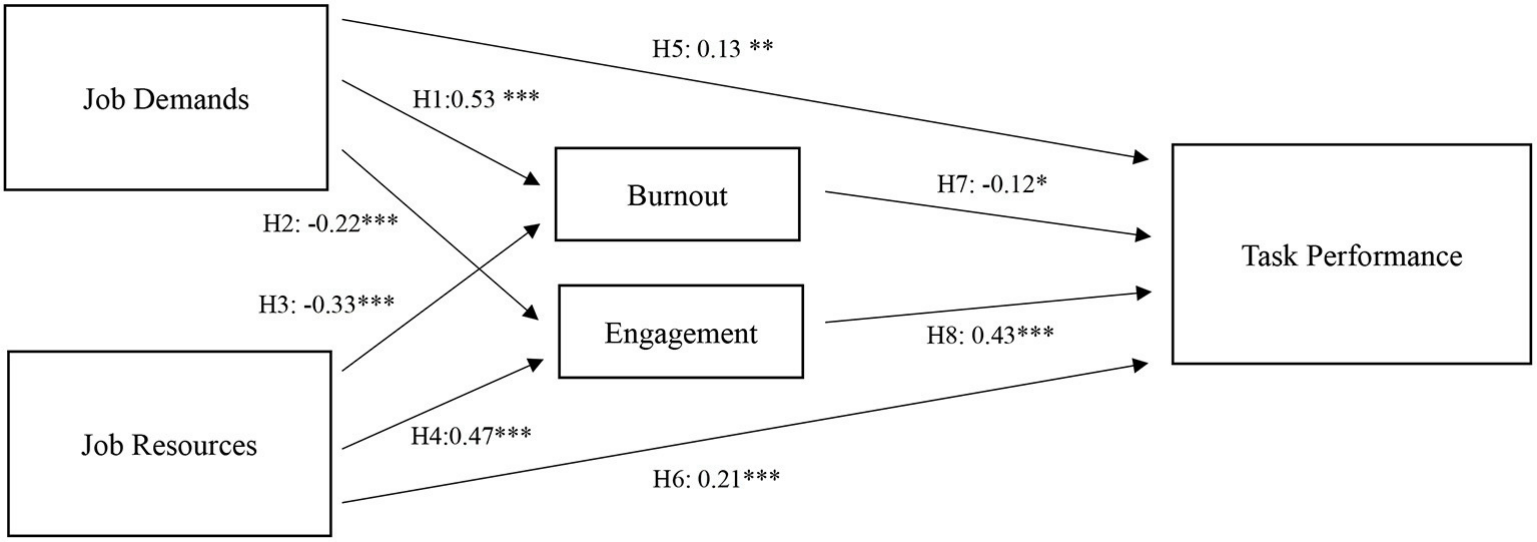
Standardized estimates of the hypothesized model. *N* = 537; *p < 0.05, **p < 0.01, ***p < 0.001.

In Figure 2, we present the standardized coefficients of the SEM model. The results show JD had positive effects on burnout (β = 0.53, *p* < 0.001) and negative effects on work engagement (β = −0.22, *p* < 0.001). These findings confirm Hypothesis 1 and 2. Meanwhile, JR were positively associated with work engagement (β = 0.47, *p* < 0.001) and negatively associated with burnout (β = −0.33, *p* < 0.001). These findings support Hypothesis 3 and 4. Conflicted with Hypothesis 5, we found JD had a positive and direct effect on task performance, though the estimate was small (β = 0.13, *p* < 0.01). JR had a medium effect on task performance (β = 0.21, *p* < 0.001) which is consistent with Hypothesis 6. Finally, burnout (β = −0.12, *p* < 0.05) and work engagement (β = 0.43, *p* < 0.001) significantly affect task performance. These findings confirm Hypothesis 7 and 8.

Finally, JD had significant and indirect effects on task performance through its effects on burnout and work engagement (β = −0.16, *p* < 0.001). Likewise, the indirect effect of JR on task performance through burnout and work engagement was also significant (β = 0.24, *p* < 0.001). These findings support Hypothesis 9 and confirm that burnout and work engagement partially mediate the associations between JD-R and task performance.

## Discussion

The sampled social workers reported high JD and JR. As shown in previous research (38, 40), JD had a strong and positive effect on burnout but a medium and negative effect on work engagement. Both of these results ordinarily lead to low task performance. However, contradicted with the JD-R Theory, we also found high JD in social workers were directly associated with positive task performance, although the size of the estimate was small (β = 0.13). It may be that social workers are more likely to increase their job performance when facing high job demands because the demands are related to helping vulnerable populations, which make the work meaningful (68). In short, though high JD may cause social workers to achieve high task performance, its effects on burnout and work engagement actually lowered task performance. Accordingly, the sum effect of JD on task performance was small and insignificant, but it considerably increased burnout and lowered work engagement amongst Chinese social workers.

In contrast, JR had a medium and negative effect on burnout plus a strong and positive effect on work engagement. Both of its effects on burnout and work engagement led to high task performances. Additionally, JR also had a medium and positive effect on task performance directly, which means it both directly and indirectly effects task performance. The findings of the SEM analysis also revealed approximately half of the effects JD-R imposed on task performance, were intervened by burnout and work engagement. This finding indicates JD and JR could be used to design new interventions that improve task performance. Such interventions could seek to reduce burnout and improve work engagement of social workers (38).

Overall, the findings of this study offer policy, managerial practice, and research implications. First, the study illuminates the prevalence of high JD amongst Chinese social workers. Given high JD pose a significant risk of burnout, which has significant impacts on public health outcomes and health service delivery for their clients (5, 6, 16), Chinese social work agencies should initiate policies such as work-life balance policy to alleviate high workloads and provide support to make emotional workloads more manageable (69–71). These policies will not only reduce social worker burnout, but they also have the potential to increase public health outcomes and health service delivery of the clients.

Second, social work agencies could impose managerial practice such as heightened supervision to encourage safer, more positive work environments (72–74). Supervision provides social workers the opportunity to engage in reflection of the emotional challenges of their jobs with someone who can provide support and help problem solve. Moreover, reflection with a supervisor can act as a JR which helps reduce the physiological costs of high emotional workloads. Currently, even though supervision has been shown to have significant effects on job-related issues, it is not widely practiced, especially in smaller cities or rural areas (75, 76).

Third, as the findings of the study demonstrate, JR have a strong effect on task performance. As such, social work agencies should consider implementing policies and managerial practices that promote collegial environments and also provide employees direct job performance feedback. Encouraging these types of positive work environments could help reduce burnout, increase engagement, and improve task performance amongst employees (35). Given social workers play a vital role in improving health care services and reducing inequities for their clients, promoting work environments of social workers would have profound effects for promoting health service delivery and public health outcomes for the vulnerable Chinese clients' social workers serve (5, 6, 16).

Fourth, given the negative effects burnout can impose on employees' mental and physical health, Chinese social work agencies should consider providing burnout-reduction services and public health interventions. For example, empirical studies have shown mindfulness-based stress reduction (MBSR), mindfulness-based cognitive therapy (MBCT), and mindfulness-based interventions (MBI) all can successfully decrease burnout, while improving mental health and wellbeing (77–79). Alternatively, emotional intelligence training has been shown to reduce job burnout (80–82). Essentially emotional intelligence training encourages individuals to understand, perceive, and utilize emotions to increase relationships and positive thinking (80, 82). Moreover, Cao et al.'s (82) recent study found individuals with higher emotional intelligence, tend to have lower rates of burnout because of their ability to better regulate inner emotions. Social work agencies in China should also consider adopting emotional intelligence training for their workers to improve emotional intelligence amongst employees to not only reduce burnout, but also improve their service delivery (81, 82). Thus, agencies should consider implementing these types of programs to reduce the extent of burnout and to improve wellbeing of their workers.

Fifth, given JD were positively associated with job performance amongst social workers in China, future studies may want to examine how the effects of JD on job performance are varied by type of employment: public, private and non-profit. It may be that social workers at non-profit organizations are more likely to increase their job performance when job demands are high than other workers due to the nature and meaningfulness of their work (68, 83–85).

Sixth, with respect to research implications, given the main tasks of social workers are centered around public health and resolving social inequities, further research should examine how the effects of social worker job burnout and work engagement effect public health service delivery and outcomes of their clients (5, 16). Understanding the mechanisms between job burnout, work engagement, task performance, and public health outcomes is crucial for an ethical and successful social work practice to adequately serve vulnerable populations in need of services and support. This knowledge has profound implications for promoting public health service delivery and outcomes.

Finally, uneven social work development across China can also hinder various agencies, especially smaller ones, from properly employing JD reduction or JR improvement practices (1, 3). Resource constraints severely limit the degree to which these agencies can improve the work conditions of their employees. Increasing governmental policies that provide supportive funds for small or rural social work agencies may have the potential to improve the labor force amongst social worker and to promote heath service delivery and public health outcomes for various vulnerable clients (5, 6, 16).

In addition, this study has theoretical implications. This study further extends the theoretical development and application of JD-R model in Chinese social workers. The study enhances the knowledge of JR as a vital intercultural construct by providing support for the relationships between JR, work engagement, and task performance in Chinese social workers. However, the validity tests in Appendix 2 suggest certain items in burnout and job demands, particularly in emotional workload and changes in tasks dimensions, may not fit Chinese social workers well. The relative average variance extracted (AVE) for these scales was between 0.40 and 0.42, suggesting low convergence validity and discriminatory validity (square root of AVE lower than inter-construct correlation) of the scales. It is important to design scales capture underline constructs while have empirically convergence and discriminatory validity (66). Future studies are warranted to develop burnout and job demands scales that better fit Chinese social workers theoretically and empirically.

Our study also had several limitations. First, our analyses were based on a cross-sectional dataset, thus the results do not consider causal relationships amongst JD-R, burnout, work engagement, and task performance. Future research may utilize longitudinal data to properly account for temporality and to test for bidirectionality. Moreover, our dataset was compiled using the participants' self-reports. Self-reporting can riddle the data with intentional and unintentional reporting errors because the participants may have underreported JD or overreported JR within their work experiences. Future studies should consider using data triangulation to overcome this type of bias.

In addition to JD and JR, there are also many factors that affect job burnout such as personal demographics, psychological characteristics, and even environmental factors such as social media and social support (86–90) that can affect participants' answers. Our study did not test for any of these variables, so this absence may have affected the reported estimates. Additionally, our data was collected from social workers concentrated solely in Guangzhou, China. Thus, these findings may not be generalizable to all social workers throughout China. A future study could expand upon our findings by exploring whether employment in rural or urban regions of China affect the mediational pathway between JD-R, burnout, work engagement, and task performance.

## Conclusion

This study analyzed data collected from 537 social workers in Guangzhou, China, to examine how JD-R affects task performance, and whether a relationship existed between burnout and work engagement amongst Chinese social workers. The study's results support previous cross-cultural research, which have shown JD-R significantly influences burnout and work engagement and that JD-R affects task performance through burnout and work engagement. Given the strong effects of JD on burnout, this study calls for public health interventions on JD of social workers in China from both government and social work agencies. Burnout out is a serious problem that not only affects the work performance of social workers but also on the health and overall wellbeing of social workers. Implementing measures to reduce JD will reduce burnout which in turn can even reduce the morbidity rates of social workers (24). Moreover, these recommended healthcare interventions will not only affect the social work labor force for the better, but they will also promote heath service delivery and public health outcomes for the vulnerable populations social workers serve.

## Data availability statement

The raw data supporting the conclusions of this article will be made available by the authors, without undue reservation.

## Ethics statement

The studies involving human participants were reviewed and approved by Review Committee, School of Public Administration Guangdong University of Foreign Studies. Written informed consent for participation was not required for this study in accordance with the national legislation and the institutional requirements.

## Author contributions

BT, XL, SS, and CH: conceptualization, validation, formal analysis, and writing—original draft preparation. BT and CH: methodology and software, resources, and investigation and data curation. All authors contributed to the article and approved the submitted version.

## Funding

This study was supported by the National Social Science Fund of China (No. 20BGL277) and Guangdong University of Foreign Studies Program (No. 19WT01).

## Conflict of interest

The authors declare that the research was conducted in the absence of any commercial or financial relationships that could be construed as a potential conflict of interest.

## Publisher's note

All claims expressed in this article are solely those of the authors and do not necessarily represent those of their affiliated organizations, or those of the publisher, the editors and the reviewers. Any product that may be evaluated in this article, or claim that may be made by its manufacturer, is not guaranteed or endorsed by the publisher.
