# Supplementary material for: Job demands, resources, and task performance in Chinese social workers: Roles of burnout and work engagement
Source: Front Public Health. 2022 Jul 19;10:908921. doi: 10.3389/fpubh.2022.908921 (PMC9343730; doi:10.3389/fpubh.2022.908921)
Supplement: Supplementary file 1 [file Data_Sheet_1.docx]

**Appendix 1: Scale Items**

| **Task Performance** |
| --- |
| 1.Achieves the objectives of the job |
| 2.Meet criteria for performance |
| 3.Demonstrates expertise in all job-related tasks |
| 4.Fulfills all the requirements of the job |
| 5.Could manage more responsibility than typically assigned |
| 6.Appears suitable for a higher-level role |
| 7.Is competent in all areas of the job, handles tasks with proficiency |
| 8.Performs well in the overall job by carrying out tasks as expected |
| 9.Plans and organizes to achieve objectives of the jobs and meet deadlines |
| **Work Engagement** |
| 1. At my work, I feel bursting with energy |
| 2. At my job, I feel strong and vigorous |
| 3. When I get up in the morning, I feel like going to work |
| 4. I am enthusiastic about my job |
| 5. My job inspires me |
| 6. I am proud on the work that I do |
| 7. I feel happy when I am working intensely |
| 8. I am immersed in my work |
| 9. I get carried away when I’m working |
| **Burnout** |
| 1.I always find new and interesting aspects in my work |
| 2.There are days when I feel tired before I arrive at work |
| 3.It happens more and more often that I talk about my work in a negative way |
| 4.After work, I tend to need more time than in the past in order to relax and feel better |
| 5.I can tolerate the pressure of my work very well |
| 6.Lately, I tend to think less at work and do my job almost mechanically |
| 7.I find my work to be a positive challenge |
| 8.During my work, I often feel emotionally drained |
| 9.Over time, one can become disconnected from this type of work |
| 10.After working, I have enough energy for my leisure activities |
| 11.Sometimes I feel sickened by my work tasks |
| 12.After my work, I usually feel worn out and weary |
| 13.This is the only type of work that I can imagine myself doing |
| 14.Usually, I can manage the amount of my work well |
| 15.I feel more and more engaged in my work |
| 16.When I work, I usually feel energized |
| **Job Demands** |
| 1.Do you have too much work to do? |
| 2.Do you have to work extra hard in order to complete something? |
| 3.Do you have to hurry? |
| 4.Would you prefer a calmer work pace? |
| 5.Does your work demand a lot from you emotionally? |
| 6.In your work, do you have to be able to convince or persuade people? |
| 7.Are you confronted with things that affect you personally in your work? |
| 8.Does your work put you in emotionally upsetting situations? |
| 9.Have the proposed changes in your tasks been introduced well? |
| 10.Do you find it difficult to adapt to changes in your tasks? |
| 11.Do the changes in your tasks cause you problems? |
| 12.Do the changes in your tasks have negative consequences for you? |
| **Job Resources** |
| 1.In your work, do you feel appreciated by your colleagues? |
| 2.Do you get on well with you colleagues? |
| 3.Can you count on your colleagues when you encounter difficulties in your work? |
| 4.Is there a good atmosphere between you and your colleagues? |
| 5.In your work, do you feel appreciated by your superior? |
| 6.Do you get on well with you superior? |
| 7.Can you count on your superior when you come across difficulties in your work? |
| 8.Is there a good atmosphere between you and your superior? |
| 9.Does your work give you the opportunity to check on how well you are doing your work? |
| 10.Does your work provide you with direct feedback on how well you are doing your work? |
| 11.Do you receive sufficient information on the results of your work? |
| 12.Does your superior inform you about how well you are doing your work? |

**Appendix 2: Reliability and Validity Tests of the Scales**

| **Variable** | **Subscale** | **Items** | **Cronbach's Alpha** | **Factor Loading** | **Average Variance Extracted** |
| --- | --- | --- | --- | --- | --- |
| Task Performance |  | 1 | 0.935 | 0.792 | 0.625 |
|  |  | 2 |  | 0.765 |  |
|  |  | 3 |  | 0.814 |  |
|  |  | 4 |  | 0.848 |  |
|  |  | 5 |  | 0.812 |  |
|  |  | 6 |  | 0.786 |  |
|  |  | 7 |  | 0.776 |  |
|  |  | 8 |  | 0.856 |  |
|  |  | 9 |  | 0.645 |  |
| Work Engagement |  | 1 | 0.949 | 0.803 | 0.689 |
|  |  | 2 |  | 0.834 |  |
|  |  | 3 |  | 0.731 |  |
|  |  | 4 |  | 0.872 |  |
|  |  | 5 |  | 0.862 |  |
|  |  | 6 |  | 0.842 |  |
|  |  | 7 |  | 0.889 |  |
|  |  | 8 |  | 0.858 |  |
|  |  | 9 |  | 0.763 |  |
| Burnout |  | 1 | 0.849 | 0.612 | 0.414 |
|  |  | 2 |  | 0.688 |  |
|  |  | 3 |  | 0.673 |  |
|  |  | 4 |  | 0.627 |  |
|  |  | 5 |  | 0.515 |  |
|  |  | 6 |  | 0.585 |  |
|  |  | 7 |  | 0.696 |  |
|  |  | 8 |  | 0.764 |  |
|  |  | 9 |  | 0.547 |  |
|  |  | 10 |  | 0.545 |  |
|  |  | 11 |  | 0.755 |  |
|  |  | 12 |  | 0.743 |  |
|  |  | 13 |  | -0.132 |  |
|  |  | 14 |  | 0.579 |  |
|  |  | 15 |  | 0.756 |  |
|  |  | 16 |  | 0.781 |  |
| Job Demands | Workload | 1 | 0.820 | 0.857 | 0.562 |
|  |  | 2 |  | 0.820 |  |
|  |  | 3 |  | 0.630 |  |
|  |  | 4 |  | 0.666 |  |
|  | Emotional Workload | 1 | 0.645 | 0.437 | 0.403 |
|  |  | 2 |  | 0.389 |  |
|  |  | 3 |  | 0.733 |  |
|  |  | 4 |  | 0.856 |  |
|  | Changes in Tasks | 1 | 0.673 | 0.156 | 0.421 |
|  |  | 2 |  | 0.549 |  |
|  |  | 3 |  | 0.847 |  |
|  |  | 4 |  | 0.799 |  |
| Job Resources | Relation w. Colleague | 1 | 0.876 | 0.823 | 0.741 |
|  |  | 2 |  | 0.901 |  |
|  |  | 3 |  | 0.802 |  |
|  |  | 4 |  | 0.910 |  |
|  | Relation w. Supervisor | 1 | 0.929 | 0.865 | 0.825 |
|  |  | 2 |  | 0.947 |  |
|  |  | 3 |  | 0.866 |  |
|  |  | 4 |  | 0.950 |  |
|  | Information Feedback | 1 | 0.869 | 0.884 | 0.772 |
|  |  | 2 |  | 0.942 |  |
|  |  | 3 |  | 0.871 |  |
|  |  | 4 |  | 0.812 |  |
